# Is COVID-19 associated with delayed treatment of road traffic injuries arriving at the emergency department?

**DOI:** 10.1186/s13104-026-07694-w

**Published:** 2026-01-31

**Authors:** Salman Muhammad Soomar, Maria Khan, F. N. U. Adnan

**Affiliations:** 1 Aga Khan University, Karachi, Pakistan; 2 Jinnah Sindh Medical University, Karachi, Pakistan; 3 Kings College Hospital London, Dubai, UAE

**Keywords:** Road Traffic Injuries, Emergency, COVID-19, Treatment delay

## Abstract

**Objective:**

This study aims to determine the delay in treatment of road traffic injuries in the ED during the COVID-19 pandemic.

**Results:**

Out of 373 RTI patients, the majority were males (312, 83.6%), and the mean ± SD age was 32.2 ± 17.4. Most injuries were fractures in the upper & lower limbs (236, 63.3%), and 302 (81.0%) underwent surgery. Of 373, 74 (19.8%) RTI patients were positive for COVID-19. The mean ± SD number of hours from ED arrival to treatment start in COVID-19 positive patients was 10.9 ± 6.7, while 6.4 ± 1.6 in negative patients. About 65% of the patients had a delay in treatment (*n* = 242). After 30 days of follow-up, 21 (5.6%) patients were dead. The adjusted odds of delay in treatment of RTI patients were 1.80 times (95% CI 1.27–2.45) in males compared to females. The adjusted odds of treatment delays in COVID-19 positive patients were 1.47 times (95% CI 1.13–1.92) compared to negative patients.

**Supplementary Information:**

The online version contains supplementary material available at 10.1186/s13104-026-07694-w.

## Introduction

 Traffic accident injuries contribute significantly to mortality & morbidity worldwide, often resulting from multiple interrelated factors. Key contributors include delays in reaching the hospital, delays in providing first aid and medical intervention, and shortages of critical healthcare resources such as hospital beds, trauma units, and operating rooms. Additionally, the severity of the accident itself plays a crucial role in determining outcomes, as does the adequacy of vehicle safety equipment. Human factors such as speeding, alcohol consumption, and driver fatigue further exacerbate injury severity and mortality [1]. Vehicle-related risks including inadequate safety features, unsafe modifications, and overloaded vehicles also increase the likelihood of severe injury and death. Environmental factors, including lack of traffic signs, poor road infrastructure, and hazardous roadside conditions, additionally influence fatality rates [1, 2].

COVID-19 pandemic has posed profound challenges to the healthcare system and its services globally. It has changed patients’ admission, referral patterns, and rehabilitation management [3]. With the surge of positive COVID-19 cases, there was a shortage of intensive care unit beds and hospital staff. Moreover, it affected emergency admissions and surgical procedures. COVID-19 testing became mandatory before admission, treatment, or any clinical procedure. Globally, hospital systems reallocated their resources to combat the high influx of COVID-19 patients. This reallocation of resources and new guidelines impacts patient treatment and is associated with a delay in the treatment of patients with road traffic injuries presenting to the Emergency Department [4, 5].

Road Traffic Injuries (RTIs) are a significant cause of worldwide morbidity and mortality. There is a huge burden of road traffic accidents in Pakistan. An estimate shows a 22% rise in RTIs since 2015, resulting in more than 40,000 deaths [6, 7]. The RTIs can include head injuries, injuries to the chest, abdomen, and pelvis, as well as traumatic injuries [8]. Injury severity is a leading factor as a cause for RTI-related fatalities.

Moreover, RTIs in Pakistan resulted in casualties due to various factors including delay in arrival to the hospital, delay in first aid and medical attention, and lack of availability of beds and trauma and operating rooms [9, 10]. The biggest challenge during the COVID-19 pandemic pre-vaccination era was waiting for a COVID-19 Polymerase Chain Reaction (PCR) test report as per the protocol and international guidelines, which was unified in the healthcare system globally caused the delay in treatment of patients with RTI as well [11, 12]. A study in Norway reported that the COVID-19 pandemic resulted in a mean ±standard deviation (SD) 6.30 (± 1.24) hours delay in treatment of COVID-positive patients with road traffic injuries and an increasing mean (± SD) length of hospital stay of 2 days (± 1.0) [13].

Similarly, a study in the United States reported delays in treating hip fractures due to an RTI in COVID-positive patients for up to 4 days [14]. The delay in treatment due to any COVID-19-related factors in RTI patients can be associated with severe health issues resulting in morbidity and mortality, causing an indirect burden on the healthcare system [15]. The delay in patient care due to the COVID-19 pandemic is seldom studied. Therefore, this study aims to observe the delay in treatment of RTIs in the ED during the COVID-19 pandemic using tertiary care hospital data.

## Methods

We conducted a prospective cohort study to understand the delay in the treatment of RTIs during the COVID-19 pandemic at the Emergency Department (ED) of a tertiary care hospital in Karachi, Pakistan. Patients with RTIs presented to the ED from April 1, 2020- February 28, 2022, tested for COVID-19 were included in the study and patients with ESI triage levels 1 and 2, existing comorbidities, severe injuries, and dead-on-arrival (DOA) were excluded. Non-probability purposive sampling was used. A total of 373 patients were enrolled in the study. Data were collected on age, sex, date and time of ED admission, type of injury, diagnosis, COVID-19 test result, surgery, number of hours from ED arrival to treatment start, length of hospital stay (days), hospital disposition [shifted to ward, discharged from ED, Leave Against Medical Advice (LAMA)] and date and time of discharge.

The outcome variable of the study was the delay in treatment of RTI (Yes/ No). Treatment delay was defined as the time that elapses from when a person first notices or recognizes a symptom or potential health problem until the time they receive definitive treatment by a health care provider [15]. Considering the cut-offs reported by Amir, A., Khalil, S., & Hoda, M. F. (2016), the delay in treatment was considered Yes if the time from ED arrival and treatment started ≥ 6 h and No if it started within 6 h of ED arrival [16]. Patients were followed for 30 days after discharge to see the clinical outcomes. Clinical outcomes include alive or dead. Informed written consent was taken from adult patients above 16 years of age. Informed written consent was taken from parents and legal guardians of patients ≤ 16 years.

Data was collected from patients on the above variables using a Performa developed specifically for this study and recorded in Microsoft Excel. The validity of tool was checked through Cronbach alpha (*r* = 0.889). The data was converted into codes to prepare for analysis using STATA 16.0. Mean± standard deviation was calculated for quantitative variables such as age, the number of hours from ED arrival to treatment start, and length of hospital stay (days) after meeting the normality assumptions. The Shapiro-Wilk test was applied for that purpose. Frequency and percentages were calculated for categorical variables such as sex, type of injury, surgery, and hospital disposition. Confounders were controlled at the design phase by restriction. Data was stratified by delay in treatment. Association of baseline and injury characteristics was checked with delay in treatment. Post- stratification Chi-square (for categorical variables) and One way ANOVA (for continuous variables) test was applied. Binary logistic regression was applied to check the association between dependent and independent variables. Crude and adjusted odds ratios with 95% confidence intervals (CI) were obtained, considering a p-value ≤ 0.05 significant.

## Results

Out of the 373 RTI patients, the majority were males (312, 83.6%), and the mean ± SD age was 32.2 ± 17.4. Most of the injuries were fractures in the upper & lower limbs [107 (28.7%) & 129 (34.6%)] followed by head and neck injuries 67 (18.0%), and 302 (81.0%) underwent surgery. Of the 373 patients, 74 (19.8%) RTI patients were positive for COVID-19, there was an average of 3.33 h delay in treatment of those positive patients. This extended delay was largely related to COVID-19 specific precautions, including the need for pre-procedural infection control measures, isolation requirements, and additional diagnostic steps, all of which prolonged the workflow compared with negative COVID-19 patients. The mean ± SD number of hours from ED arrival to treatment start in COVID-19 positive patients was 10.9 ± 6.7 h, whereas in COVID-19 negative patients it was 6.4 ± 1.6 h. About 70% of the total patients had a delay in treatment (242, 64.88%). Most of the RTI patients were admitted to the ward (327, 87.7%), while 13 (3.5%) were admitted to the ICU. The mean ± SD length of hospital stay for COVID-positive patients was 11.0 ± 5.0 days. After 30 days of follow-up, 21 (5.6%) patients were dead and 352 (94.4%) were alive. The majority died due to other reasons (*n* = 14 (66.7%)). Only 3 (14.3%) were dead due to a delay in treatment (Table 1).

**Fig. 1:**
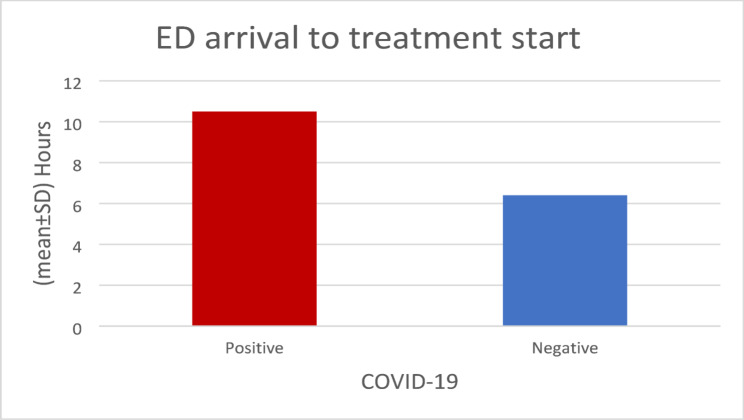
Mean time arriving at ED and treatment start in hours

The mean number of hours for the delay in the treatment for COVID-19 patients, both positive and negative, is displayed in Fig. 1.

Except for type of injury no baseline and injury characteristics were significantly associated with delay in treatment (*p* = 0.029) (Table 2).

**Table 1:** Baseline and injury characteristics for the delay in treatment of RTI patients with COVID-19 (*n* = 373).

Characteristic	*n* (%)
Age – years (mean ± SD)	32.2 ± 17.4
Sex	
Male	312 (83.6)
Female	61 (16.4)
Type of injury	
Head & neck	67 (18.0)
Upper limbs	107 (28.7)
Lower limbs	129 (34.6)
Abdomen	48 (12.9)
Other	22 (5.9)
COVID-19 test	
Negative	299 (80.2)
Positive	74 (19.8)
ED arrival to treatment start - hours (mean ± SD)	
COVID-19 positive	10.5 ± 8.9
COVID-19 negative	6.4 ± 1.6
Delay in treatment	
No	131 (35.12)
Yes	242 (64.88)
Surgery	
No	71 (19.0)
Yes	302 (81.0)
Hospital Disposition	
Ward	327 (87.7)
ICU	13 (3.5)
Discharged from ED	25 (6.7)
LAMA	8 (2.1)
Length of hospital stay – days (mean ± SD)	
COVID-19 positive	11.0 ± 5.0
COVID-19 negative	5.4 ± 3.1
30 day follow-up outcome	
Alive	352 (94.4)
Dead	21 (5.6)

*ED* emergency department, *ICU* intensive care unit, *LAMA* leave against medical advice.

**Table 2:** Baseline and injury characteristics stratified on delay in treatment of RTI patients with COVID-19 (*n* = 373)

Characteristic	Delay in treatment *n* (%)		*p*-value
	Without delayed treatment *n* = 114	With delayed treatment *n* = 259	
Age – years (mean ± SD)	31.26 ± 17.82	32.71 ± 17.14	0.613*
Sex			
Male	112 (85.50)	200 (82.64)	0.477**
Female	19 (14.50)	42 (17.36)	
Type of injury			
Head & neck	34 (25.95)	33 (13.64)	0.029**
Upper limbs	35 (26.72)	72 (29.75)	
Lower limbs	44 (33.59)	85 (35.12)	
Abdomen	11 (8.40)	37 (15.29)	
Other	7 (5.34)	15 (6.20)	
COVID-19 test			
Negative	106 (80.92)	193 (79.75)	0.788**
Positive	25 (19.08)	49 (20.25)	

*ANOVA **Chi-Square test

The multivariable analysis revealed that the adjusted odds of delay in treatment for RTI patients were 1.80 times (95% CI, 1.27–2.45) higher in males compared to females. The adjusted odds of treatment delays in COVID-19-positive patients were 1.47 times (95% CI 1.13–1.92) higher compared to COVID-19-negative patients. The delay in treatment of RTI patients resulted in 2.06 times (95% CI 1.44–2.92) higher odds for increasing the length of hospital stay in COVID-positive patients compared to COVID-19 negative patients (Table 3).

**Table 3:** Univariate & multivariable analysis reporting crude and adjusted odds ratio for delay in treatment of RTI patients with COVID-19 (*n* = 373)

Characteristic	COR (95% CI)	*p*-value	AOR (95% CI)	*p*-value
Age (years)	1.76 (1.24–1.99)	0.365	-	
Sex				
Female	1			
Male	1.29 (1.22–1.82)	0.001	1.80 (1.27–2.45)	0.003*
Type of injury				
Head & neck	1			
Upper limbs	2.96 (0.97–3.33)			
Lower limbs	1.71 (0.78-2.00)			
Abdomen	1.38 (0.95–1.86)			
Other	1.43 (1.05–1.96)	0.298	-	-
Surgery				
No	1			
Yes	1.65 (0.87–1.88)	0.665	-	-
COVID-19 test				
Negative	1		1	
Positive	1.23 (1.04–1.56)	0.011	1.47 (1.13–1.92)	0.008*
Hospital Disposition				
Ward	1			
ICU	1.16 (0.89–1.95)	0.451		
Discharged from ED	0.91 (0.33–1.11)		-	-
LAMA	0.62 (0.43–1.20)			
Length of hospital stay (days)	1.93 (1.67–2.45)	0.005	2.06 (1.44–2.92)	< 0.001*

*Significant at Multivariable level

*ED* emergency department, *ICU* intensive care unit, *LAMA* leave against medical advice

## Discussion

The risk of COVID-19 infection in patients presenting to the ER has led to the implementation of institutional protocols that mandate COVID-19 testing in all patients. This test results in a delay of definitive treatment but does effectively reduce the risk of hospital-acquired diseases in other patients and hospital personnel. Most of the affected individuals were males (312, 83.6%), and mostly younger in age, the mean age was 32 years. The adjusted odds of delay in treatment for RTI patients were 1.80 times higher in males. Similar findings were reported by González-Sánchez, Guadalupe, et al. (2021) that males had the highest risk of RTI (RR = 1.82), and those mostly in young age groups [17].

Literature mentions that in severe cases, emergency surgery is necessary to save the lives of victims of deadly accidents [18]. In this study most of the injuries were fractures and 302 (81.0%) underwent surgery. In a study from Ethiopia reported that 82% of the RTI cases underwent surgery and admitted to surgical and orthopedic ward post surgeries [19]. Verlaan, J. J., et al. (2004) mentioned that depending on the kind and location of trauma, different surgical procedures are used to treat fractures and injuries. In order to stop neurological decline, head and neck injuries such traumatic brain injuries, skull fractures, and cervical spine fractures sometimes call for immediate surgical operations like craniotomy, decompression, or spinal stabilization [20]. Erdem, T. E., & Çolak, T. S. (2025) also mentioned regarding the surgical interventions; when upper limb fractures, such as those of the wrist, ulna, radius, and humerus, are displaced or unstable, surgical fixation is usually required, along with further treatment for soft tissue injuries such nerve or vascular injury. Due to their weight-bearing function and intricacy, lower limb fractures including the femur, tibia, fibula, and ankle often require surgical intervention. Procedures range from internal fixation to joint reconstruction to restore function and allow for early mobilization. Due to the high risk of morbidity, abdominal injuries affecting solid organs, hollow viscera, or vascular structures frequently necessitate emergency surgery for organ repair and hemorrhage control, particularly when diagnosis or treatment is delayed [21]. Furthermore, surgical interventions are required to control bleeding, restore stability, and encourage healing, other injuries such as chest trauma, pelvic fractures, and soft tissue injury may require surgical stabilization or reconstructive surgery [22]. Injury severity, displacement, related complications, and treatment timing all have an impact on the choice of surgical intervention across all injury types, underscoring the crucial role that prompt and effective operative management has in maximizing patient outcomes [23].

This study showed a 1.5 times chance of delay in definitive management in patients who tested positive for COVID-19 when compared to those who tested negative. Delay in management resulted in nearly two times the odds of a prolonged hospital stay. A study conducted in Norway also supports the results of the current study, as a delay of 6.30 (± 1.24) hours was seen with patients of RTAs that had a positive COVID-19 test [13].

Point-of-care testing for COVID-19 was rapidly implemented in emergency departments worldwide to isolate COVID-19-positive patients, prevent hospital-acquired SARS-CoV-2 infection, protect healthcare workers, and improve patient flows [24]. While clear benefits in isolation precautions exist, there are many pitfalls such as added costs, materials, time, and, as demonstrated by our study, a possible unacceptable delay in definitive treatment. Such data is crucial when considering national and institutional guidelines for point-of-care testing to standardize practices and improve patient outcomes across the country [24].

While our study shows that testing positive for COVID-19 resulted in a delay in treatment, literature from around the world is not specific. A survey by Serra-Torres et al. showed that, when comparing patients admitted during the COVID-19 period to those admitted before it, patients admitted during the COVID-19 pandemic presented to the hospital significantly later. However, the time between presenting to the ER and surgical management remained the same, and the length of hospital stay did not increased [14].

This study found that as many as 19.8% of patients were COVID-19 positive. Hence, many patients had a delay in management due to a positive test. This decreases the quality of care while also raising levels of morbidity and mortality. Overall, delays in patient treatment are associated with worse patient outcomes. While COVID-19 testing is essential during the peak incidences of the pandemic, effective, sustainable, and efficient solutions must be implemented to not compromise patient care while also limiting the spread of the virus. Delays in trauma care might raise treatment complexity and total expenses in Pakistan’s primarily out-of-pocket healthcare system. Patients may be more financially vulnerable as a result, and prompt treatment is linked to both clinical and financial results [25, 26]. This can lead to impoverishment due to the high cost of surgical management that is often required and catastrophic expenditure on healthcare [27]. In a lower-middle-income country like Pakistan, this is all too common and must be carefully avoided via institutional guidelines to minimize costs.

## Strengths and limitations

This study has some limitations. This study was conducted at a single tertiary care hospital in Karachi, Pakistan with a small sample size and diversity of presentations therefore the findings were not representative of other hospitals or regions and lacked generalizability. There is a need for further research with a larger sample size and more diverse samples to validate the results. Our retrospective approach poses biases in data collection. This study focuses on patients who were presented to the ED and were screened for COVID-19 and also included patients who were alive at the time of presentation to the ED to receive care in the ED. This could limit the number of documented patients or cause a selection bias. Moreover, the inherent selection bias of non-probability purposive sampling cannot be excluded. Furthermore, comparison with a pre-COVID-19 period is beyond our scope due to limited resources. However, such a comparison can help assess an overall delay in definitive treatment before COVID-19 was a factor and not solely when patients test negative for COVID-19.

A major limitation is that this study does not consider potential delays that may occur before a patient arrives at the hospital, including delays in emergency medical services.

Despite these limitations, our study is the first in the region, to present foundational work on the delay associated with COVID-19 testing and its effects on the length of hospital stay. When considering protocol for RTI patients in the ER, this can be factored in. Our sample size, though from a single center, is like many other studies.

## Conclusion

Significant treatment delays for RTI patients during the COVID-19 pandemic are highlighted in this study, especially for males and those who tested positive for the virus. Nearly 70% of patients had delayed treatment, with COVID-19-positive patients waiting significantly longer from ED entrance to treatment beginning, even though most patients suffered limb fractures and needed surgical intervention. Longer hospital stays were also linked to delays, particularly among those who tested positive for COVID-19. Only a small percentage of deaths were directly linked to delayed care, even if total 30-day mortality remained low. The results highlight the necessity of enhanced emergency response protocols, better triage systems, and focused tactics to reduce treatment delays during infectious disease outbreaks, guaranteeing prompt and fair care for trauma patients.

## Supplementary Information

Supplementary material 1.

## Data Availability

Data and materials are available with the corresponding author on reasonable request.

## Abbreviations

ED: Emergency department
ICU: Intensive care unit
LAMA: Leave against medical advice
PCR: Polymerase chain reaction
RTI: Road traffic injuries
SD: Standard deviation
