# Supplementary material for: Is COVID-19 associated with delayed treatment of road traffic injuries arriving at the emergency department?
Source: BMC Res Notes. 2026 Jan 31;19:95. doi: 10.1186/s13104-026-07694-w (PMC12947434; doi:10.1186/s13104-026-07694-w)
Supplement: Supplementary file 1 — Supplementary material 1. [file 13104_2026_7694_MOESM1_ESM.pdf]

Is COVID-19 associated with delayed treatment of Road Traffic Injuries arriving at the Emergency Department?

Questionnaire

**Question**	**Code**	**Response**
Medical Record Number	Number	
Age	In years	
Sex	Male<br>Female	
Type of injury	Head & neck<br>Upper limbs<br>Lower limbs<br>Abdomen<br>Other	
COVID-19 test	Negative<br>Positive	
ED arrival to treatment start	In hours	
Delay in treatment	No<br>Yes	
Surgery	No<br>Yes	
Hospital Disposition	Ward<br>ICU<br>Discharged from ED<br>LAMA	
Length of hospital stay	In days	
Outcome	Alive<br>Dead	
